# Understanding and overcoming geographical barriers for scaling up dog vaccinations against rabies

**DOI:** 10.1038/s41598-024-82085-4

**Published:** 2024-12-28

**Authors:** Maganga Sambo, Katie Hampson, Paul C. D. Johnson, Olatunji O. Johnson

**Affiliations:** 1 Environmental Health and Ecological Sciences Department, Ifakara Health Institute, Ifakara, Tanzania; 2 Boyd Orr Centre for Population and Ecosystem Health, School of Biodiversity, One Health and Veterinary Medicine, University of Glasgow, Glasgow, UK; 3 Department of Mathematics, Manchester University, Manchester, UK

**Keywords:** Canine rabies, Mass dog vaccination, Coverage, Geospatial, Campaign completeness, Ecology, Environmental sciences

## Abstract

Rabies causes 59,000 human deaths annually in over 150 countries. Mass dog vaccination (MDV) is key to controlling dog rabies, requiring 70% coverage in the susceptible dog population to eliminate rabies deaths. MDV campaigns must achieve geographical homogeneity of coverage. Although rabies elimination is feasible, operation challenges exist, especially in hard-to-reach areas. We conducted geospatial modelling to identify geographical factors affecting MDV success in terms of campaign completeness and vaccination coverage across 25 districts in south-eastern Tanzania. From October 2016 to January 2017, about 81,000 dogs were vaccinated in 1,379 (68%) villages within these districts. Multivariable regression analysis revealed that land cover, residence, poverty, and elevation were associated with campaign completeness. The odds of achieving completeness in croplands were 1.75 times higher compared to forests. Vaccination coverage was influenced by residence, area, poverty index, and elevation, with urban areas having lower odds of achieving coverage compared to rural areas. Coverage probabilities exceeding 70% were lower on islands, highlands, urban areas, and areas bordering protected areas. As the 2030 deadline for "zero dog-mediated human rabies deaths" approaches, operational and logistical challenges in MDV campaigns persist. Our data provide insights into MDV success and failure, guiding future control efforts to improve their effectiveness.

## Introduction

Rabies causes approximately 59,000 human deaths annually across the world, with over 95% of the burden falling in Africa and Asia and over 98% from bites from rabid domestic dogs^1^. Although rabies is a fatal disease once clinical signs appear, but it is entirely preventable through three key interventions. The first intervention is raising awareness about rabies, which empowers communities to prevent the disease in animals and seek timely care following potential exposure^1^. The second is post-exposure prophylaxis (PEP), a treatment that includes a series of rabies vaccines and, in some cases, rabies immunoglobulin (RIG). When administered promptly after exposure, PEP is nearly 100% effective in preventing human rabies. The third intervention is mass dog vaccination (MDV), which targets the source of the virus—dogs, responsible for about 98% human rabies cases^1^. Achieving and maintaining 70% vaccination coverage in susceptible dog populations is sufficient to halt rabies transmission^2^.

While human rabies can be effectively prevented with PEP, this intervention is costly and often inaccessible to people in remote and impoverished communities^3,4^. Consequently, many individuals die due to lack of access to life-saving PEP^1,4^. For example, a case study in Tanzania estimated that a patient in a rural area, where most people live on less than US$1.25 per day, would need to spend over US$100 to access and complete the World Health Organization’s (WHO) recommended PEP regimen^3^. These economic and logistical barriers lead to poor compliance with PEP, delays in seeking treatment, and an increased risk of death^4,5^.

Given the challenges associated with PEP, MDV is a more cost-effective approach to eliminating human rabies by targeting its primary reservoir—the domestic dog^1,5^. The cost per dog vaccinated is typically under US$7, and although MDV campaigns require upfront investment, the long-term benefits include reductions in human rabies cases and the subsequent need for PEP, making MDV a highly cost-effective strategy^4,6^. Over time, successful implementation of MDV could lead to the eventual elimination of the disease, resulting in significant reductions in healthcare costs^7^.

Mass dog vaccination has eliminated dog-mediated rabies from high-income countries and much of the Latin America and Caribbean^8^. Research has demonstrated that parenteral MDVs are the most effective approach for addressing dog-mediated rabies on a large scale in African countries where dogs are free-roaming^9,10^. In Tanzania, although over 78% of owned dogs are free-roaming^11^, these dogs are still accessible for parenteral vaccination, ensuring that the critical 70% coverage required for effective rabies control can be achieved^12^. In line with the United Nations Sustainable Development Goals, the leadership of the WHO, the World Organization for Animal Health (WOAH), the Food and Agriculture Organization of the United Nations (FAO) and the Global Alliance for Rabies Control (GARC) has developed a Global Strategic Plan (GSP) and has announce the formation of the United Against Rabies Forum (UARF) to provide an enabling environment for worldwide elimination of human dog-mediated rabies by 2030^13^.

The GSP states that mass dog vaccination is a proven, cost-effective way to save human lives by stopping transmission of rabies at its source. Consequently, more than one hundred countries in which rabies is currently endemic will be required to scale up MDV over the next five years ^14^. However, scaling up MDVs across rural landscapes presents logistical challenges^15^, which must be addressed to achieve and maintain herd immunity^15^. Consequently, many rabies endemic countries, particularly in sub-Saharan Africa, have either not been implemented or have only been locally implemented and achieved low dog vaccination coverage^1,16^.

To eliminate dog rabies, it is crucial to ensure that vaccination efforts reach all dog-owning communities (campaign completeness)^17,18^ and ensure at least 70% of the susceptible dog population are to interrupt transmission^2^. Mathematical modeling studies indicate that achieving this level of coverage can prevent rabies outbreaks, given the low basic reproduction number (R_0_) for or rabies, which ranges from 1.6 to 2.3^19^. Maintaining adequate herd immunity, especially among free-roaming dogs, is essential due to their high population turnover^15^. Vaccination coverage declines over time as vaccinated dogs die, new unvaccinated dogs are born, and vaccine-induced immunity wanes^15^. In our case, we used Nobivac® the dog vaccine which providing three years immunity. Therefore, when the coverage of 70% is achieved, subsequent campaigns should be conducted within 12 months to maintain herd immunity^15,19^.

To overcome MDV delivery constraints, careful micro-planning of dog vaccination campaigns is necessary. While studies have explored the social and economic determinants of dog vaccination coverage, they often neglect the influence of geographical, operational and demographic factors on campaign completeness and dog vaccination coverage. This study aims to investigate this research gap by assessing spatial heterogeneity and geographical determinants of dog vaccination campaign completeness and vaccination coverage using geospatial methods. By incorporating these geographical factors into micro planning, rabies national program managers in low- and middle-income countries can design dog vaccination interventions that bridge coverage gaps, leading to a rabies-free and healthier community.

## Methods

**Fig. 1:**
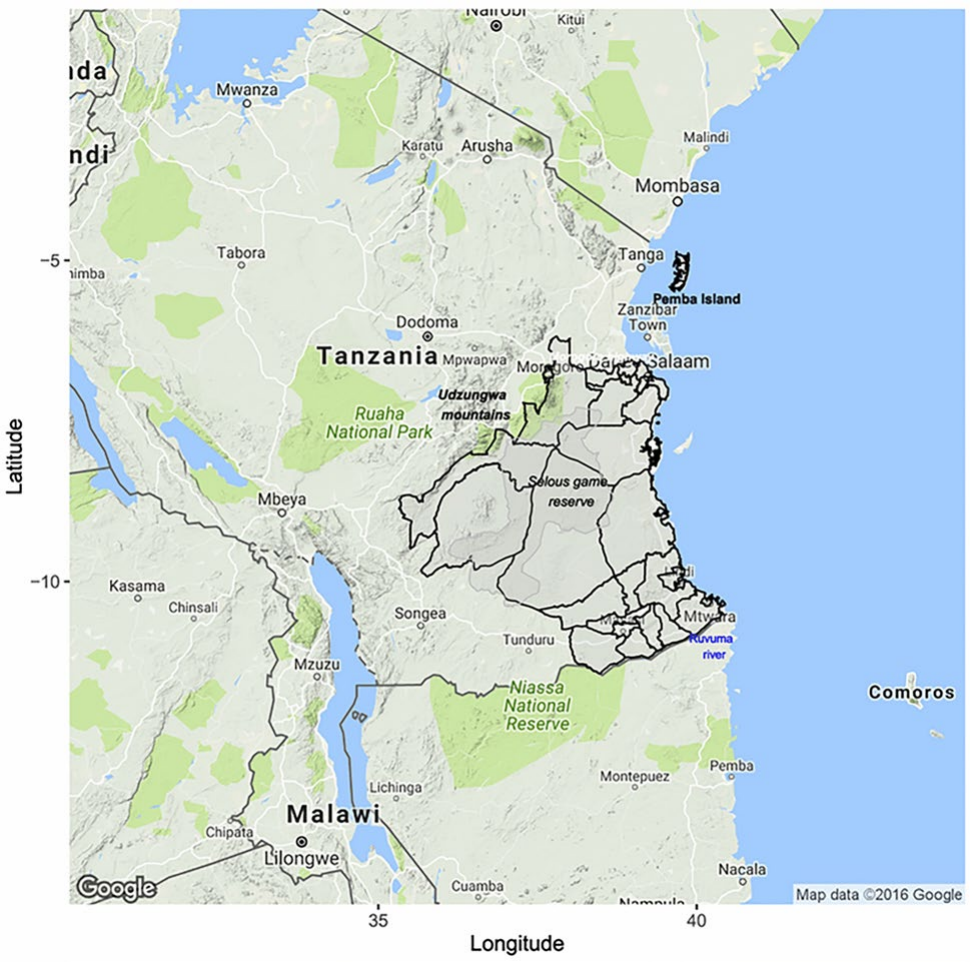
The map of Tanzania showing the study districts (demarcated in bold black boundaries) where mass dog vaccinations and post-vaccination transects were undertaken. The shapefiles used in this figure were downloaded from the Diva GIS data portal (https://www.diva-gis.org/).

We collected MDV records and data to assess coverage achieved across 25 districts in south-eastern Tanzania in 2017 as summarized in Fig. 1. We used geospatial models to assess the relationship between mass dog vaccination versus campaign completeness and dog vaccination coverage across 25 districts in south-eastern Tanzania as summarized below.

### Study sites

The study was conducted in 25 districts from five regions from south-eastern Tanzania: Lindi, Mtwara, Pwani, Dar es Salaam and Morogoro (Fig. 1 and Table 1). In Morogoro region specifically, MDV began in 2007 in the Ulanga district and in 2008 in the Kilombero district. Whereas in Dar Es Salaam region started their MDV programs in 2010 together with two districts from Morogoro region (Morogoro Rural and Morogoro urban), while the remaining districts began in 2011. In this study, Ulanga and Kilombero are referred to as districts with experience in delivering MDV since they started earlier than the others. The districts comprise rural, coastal and urban settlements, and cover an area of 160,000 km^2^. According to the official population census conducted in 2012, these districts had a population of about 8.5 million people, with an average annual population growth rate of 2.16%. Over 65% of Tanzanians live in rural areas, depending solely on agriculture and fishing for their livelihood ^20^. Those from urban areas are mostly engaged in the civil service and small or large-scale business.

**Table 1:** Characteristics of the study districts.

Re Region	District	Location	Area (km^2^**)**	No. villagesbordered toprotected areas	No. villages	Village with M MDVs	No. of vvaccinated dogs va	Estimated do dogs	Human pop p	Mean distance from HQ to VUs (SD)	Budget allocated (TZS 000,000’) (budget/no.villages in the district)
Dar Es Salaam	Ilala	Coastal-urban	365	0	26	22	4,419	8,179	1,220,611	10.5 (9.3)	12.7 (0.49)
Dar Es Salaam	Kinondoni	Coastal-urban	538	0	34	20	3,421	8,618	1,775,049	9.6 (8.7)	12.3 (0.36)
Dar Es Salaam	Temeke	Coastal-urban	728	0	30	18	3,316	5,819	1,368,881	8.1 (8.8)	12.5 (0.42)
Lindi	Kilwa	Coastal-rural	14,988	2	100	45	2,651	4,961	190,744	56.5 (26.5)	8 ( 0.08)
Lindi	Lindi Rural	Coastal-rural	5,971	0	133	120	1,640	2,788	194,143	43.9 (14.7)	8.6 (0.06)
Lindi	Lindi Urban	Coastal-urban	1,063	0	18	15	1,071	1,876	78,841	7.08 (7.8)	6.8 (0.38)
Lindi	Liwale	Inland-rural	34,301	9	76	58	431	2,386	91,380	30.9 (26.9)	7.2 (0.09)
Lindi	Nachingwea	Inland-rural	5,970	0	118	52	2,360	4,477	178,464	23.8 (23)	8.5 (0.07)
Lindi	Ruangwa	Inland-rural	2,514	0	89	74	1,643	2,095	131,080	22.5 (12.2)	8.2 (0.09)
Morogoro	Kilombero	Inland-rural	13,561	12	80	59	12,408	27,599	407,880	62.5 (49.8)	12.3 (0.15)
Morogoro	Morogoro Rural	Inland-rural	12,453	0	140	94	6,629	12,292	286,248	41.7 (19.6)	11.8 (0.08)
Morogoro	Morogoro Urban	Inland-urban	288	0	19	19	8,113	17,476	315,866	3.7 (3.2)	11.8 (0.62)
Morogoro	Ulanga	Inland-rural	23,484	14	65	63	10,141	19,256	265,203	44.8 (27.9)	12 (0.18)
Mtwara	Masasi	Inland-rural	4,002	0	147	48	1,937	5,027	247,993	36.2 (14.6)	5.7 (0.04)
Mtwara	Masasi urban	Inland-urban	753	0	12	12	874	4,504	102,696	7.3 (5.6)	5.7 (0.48)
Mtwara	Mtwara Rural	Coastal-rural	3,690	0	156	74	869	2,635	228,003	38.2 (18)	8.2 (0.05)
Mtwara	Mtwara Urban	Coastal-urban	170	0	15	15	565	1,160	108,299	5.7 (3)	5.8 (0.39)
Mtwara	Nanyumbu	Inland-rural	5,200	0	89	55	1,163	2,364	150,857	32.8 (13.5)	6.7 (0.08)
Mtwara	Newala	Inland-rural	1,951	0	153	63	702	3,630	205,492	23.2 (12)	7.7 (0.05)
Mtwara	Tandahimba	Inland-rural	2,047	0	155	126	801	1,269	227,514	20.4 (11.3)	7.3 (0.05)
Pwani	Kibaha Rural	Inland-rural	1,498	0	50	33	4,344	9,327	70,209	35.9 (18.6)	10.2 (0.20)
Pwani	Kibaha Urban	Inland-urban	705	0	53	53	4,242	6,596	128,488	13.7 (8.5)	10.3 (0.19)
Pwani	Kisarawe	Inland-rural	5,027	2	77	55	3,116	5,010	101,598	45.2 (23.1)	10.4 (0.14)
Pwani	Mkuranga	Coastal-rural	2,825	0	116	105	2,451	3,891	222,921	27 (13.3)	9.8 (0.08)
Pwani	Rufiji	Coastal-rural	12,739	8	115	99	1,920	3,128	217,274	45.9 (19.3)	10.5 (0.09)

*TZS* Tanzanian shillings, *MDV* mass dog vaccinations, *VU* vaccination unit corresponds to village in rural districts or ward in urban districts, *HQ* district headquarters, *SD* standard deviation, *No* number and *Km* kilometres. Exchange rate TZS to USD is 1 TZS = 0.000392761 USD.

The straight-line distance between the district headquarters (where the district livestock office are located) and vaccination units were calculated from their centroids. Data on whether village was bordered to protected areas were obtained from Diva GIS data portal (https://www.diva-gis.org/), number of vaccinated dogs and budget allocated for MDVs were obtained from campaign administrative logs, number of estimated dogs obtained from Sambo et al.21 whereas the rest of data were obtained from Tanzania Population and Housing Census 2012.

## Data collection

### Mass dog vaccination

Five rounds of MDV campaigns were carried out in the study districts between January 2010 and January 2017^15^. Each round of implementing dog vaccinations, campaign completeness increased, with a relatively stable completeness observed from the fourth to the fifth round^15^. The fifth campaign, conducted between October 2016 and January 2017, boasted superior data quality in terms of campaign completeness compared to earlier rounds^15^. Given the improved vaccination data for the fifth round, in this study we opted to evaluate the fifth (last) round of vaccinations. Vaccinations were conducted by teams of vaccinators traveling from village to village hosting day-long clinics at a central point to which owners voluntarily bring their dogs^15^. Vaccination teams generally consist of a vaccinator, a recorder who documents the details of vaccinated dogs into the dog registers, and a village leader to assist with vaccination set up and guiding dog owners on the vaccination process. Vaccination campaigns were publicized locally one week in advance with a reminder using loudspeakers the day before. Communities were informed about the campaign (free dog vaccinations, place, time and the day of the campaign) using loud-speakers, fliers, announcements at schools and using community messengers urging dog owners to bring their dogs for vaccinations^15^. At the vaccination points, all dogs that were vaccinated were fitted (marked) with temporary fabric collars around their necks for the temporary identification of vaccinated (marked) dogs during post-vaccination transects^22^. Vaccinations generally began at 7 A.M. in the morning and lasted until 3.30 PM in the afternoon^15^.

### Monitoring and evaluation

Immediately after vaccinations, post-vaccination evaluations using transect surveys (transects) were conducted to assess the effective of the campaigns^22^. Transects were walked (or occasionally cycled) on the same day as the campaigns from 4 to 6 p.m. when dogs were likely to be more active and visible, counting all marked (vaccinated) and unmarked (unvaccinated) dogs. These counts were then used to calculate the vaccination coverage. In rural communities, transects were conducted in two randomly selected sub-villages in each vaccinated village (villages ranged in size from two to ten sub-villages, with a median of 4 sub-villages/village), aiming to representatively sample coverage within each village^15^. In the first sub-village, enumerators were instructed to start transects at the center of the sub-village heading to the outskirts, while in the other sub-village, transects started from the edge of the sub-village and headed toward the center. Each transect was conducted by one enumerator for 1 h, therefore, taking a total of 2 h to complete each village. In urban areas, enumerators were required to cover the jurisdiction of a street (a geographical area defined from the National Census, which covers a neighborhood with several roads). To avoid bias in selecting a preferred route, enumerators determined the direction at the start of transects, at the border of sub-villages/streets and at road junctions by spinning a pen. The direction in which the pen pointed guided the enumerators’ route selection, ensuring a random choice^15^. Prior to transects, training sessions were held with enumerators who were recruited from the community by village leaders. The recruited enumerators were respected and trustworthy members of the community who were familiar with village boundaries and have basic literacy and numeracy skills. Printed protocols and data collection forms were provided to enumerators during this training. Transect data were used for calculating vaccination coverage^22^.

### Geographical and demographic factors

We considered several geographical variables including environmental, socio-economic and demographic variables that we hypothesized might influence campaign completeness and coverage, summarized in Table 2.

**Table 2:** Environmental and demographical data hypothesized to be associated with campaign completeness and coverage.

Predictor	Data type	Definition/description	Source
Area (in sq.km)	Continuous	Area of a place (i.e. village or district) including water bodies	Tanzania’s National Bureau of Statistics
Presence of protected areas	Categorical (two levels: 1 = Yes and 2 = no)	Villages bordered to forest reserves or wildlife-protected areas that are not inhabited by humans	Tanzania’s shape files for protected areas from ^20^
Elevation	Continuous	Altitude describing height above the sea level	WorldPop ^23^
Land use	Categorical (five levels: 1 = Forest, 2 = Shrubland & savannas, 3 = Croplands (including vegetation), 4 = Wetlands, 5 = Urban and build-up lands	Human-environmental interaction (human’s use or modification of the land)	MODIS ^24^
Dog density	Continuous	Number of dogs per square kilometer (excluding protected areas and water bodies)	Estimated by Sambo et al.^21^
Poverty level	Continuous	Proportion of people living in poverty, as defined by the multidimensional poverty index (takes values between 0 and 1. Whereas the higher the value the poorer the area)	WorldPop^25^
Human population density	Continuous	Number of people per square kilometer	WorldPop^26^
Residence	Categorical (two levels: 1 = Rural and 2 = Urban)	Rural is a human settlement with fewer and more sparsely distributed homes, with low human population density < 250 persons/km^2^ and reliance on agricultural income sources whereas urban is the opposite	^20^
Mean distance from district headquarters to the vaccination units	Continuous	The straight-line distance between the vaccination unit and district headquarters (where the district livestock office are located) calculated from their centroids	Tanzania’s shape files from the National Bureau of Statistics^20^

### Response variables

Our response variables in this study are vaccination campaign completeness and vaccination coverage (expressed in percentage). Vaccination completeness was measured as the proportion of vaccination units within each district where campaigns were conducted during the most recent (fifth) round of vaccination (vaccination unit corresponds to village in rural districts and ward in urban districts). For vaccination coverage, we employed different estimation methods depending on the availability of transect data for each vaccination unit. When transect data were available, we directly estimated coverage by dividing the number of vaccinated (collared) dogs by the total number of dogs observed during the surveys, regardless of whether they had collars or not. In cases where transect data were not available, we resorted to indirect estimation^15^. Indirect estimates of coverage were calculated by dividing the number of dogs vaccinated by the estimated dog population specific to each vaccination unit^21^.

### Statistical modelling

To analyze the relationship between the response variables (completeness and coverage) and the geographical and demographic factors (that were summarized in Table 2 were are used as explanatory variables), a binomial regression model was employed. The model is of the form:

$$log\left(\frac{p}{1-p}\right)=X\beta$$

where *p* is the vaccination completeness or vaccination coverage, and X is the design matrix of the geographical factors with associated regression coefficient $\beta$.

To avoid unreliable regression coefficient estimates, an assessment of multicollinearity among the predictor variables was conducted by checking the variance inflation factor (VIF). Covariates with VIF < 5 were included in the final fitting models^27^. To identify the most relevant predictors for the binomial regression model, a best subset selection approach was used. The best subset selection method evaluates all possible combinations of predictors and selects the subset of predictors that provides the best fit to the data. We evaluated all possible models and selected the model with the best fit based on Akaike Information Criteria (AIC). The regression coefficients and corresponding confidence interval of the best fit model were calculated to assess the significance of the predictor variables. If the confidence interval of exp(β) excludes 1, it indicates that the result is statistically significant.

### Geospatial modelling

To incorporate the spatial aspect of the data and account for spatial correlation, a geospatial model was fitted using a spatial binomial mixed effect model. The model is of the form:

$$log\left(\frac{p}{1-p}\right)=X\beta +S$$

where p is the vaccination completeness or vaccination coverage, X is the design matrix of geographical factors with associated regression coefficient $\beta$ and $S$ is the spatial random effect. The geospatial model incorporates a spatial random effect into the non-spatial model and aimed to capture the spatial variation in the response variable beyond what is explained by the predictor variables. Several spatial random effect structures were considered in the modeling process. These included independent random effect which assumes that the spatial random effect is independent across locations; Gaussian Markov Random Field (GMRF) structures: Besag’s improper^28,29^, as well as Leroux^30^; and Gaussian Random Field (GRF)^31^. All the models were implemented in the R-INLA package. To use the GRF, we assume that the data locations correspond to the centroid of each district or vaccination unit. To determine the most appropriate spatial random effect structure, the models were compared using the deviance information criterion (DIC). After comparing the geospatial models, it was found that the Leroux structure provided the most robust fit for our data. Therefore, only the results from the geospatial model with the Leroux structure were presented in the results section. Results from the other spatial random effect structures were included in the Appendix. The precision matrix of the Leroux structure is a combination of an identity matrix I, representing unstructured spatial effects, and the precision matrix of an intrinsic conditional autoregressive (CAR) model Q, representing structured spatial effects: $\left(1-\lambda \right)I+\lambda Q$. The parameter $\lambda$ takes the value between 0 and 1, and governs the degree of spatial structure present in the data. Lower values of $\lambda$ signify a non-spatial pattern, whereas higher values indicate a pronounced spatial pattern.

We used exceedance probability to identify areas where vaccination coverage could be improved, a concept previously used to identify hotspots and coldspots^32^. Specifically, we map the probability that vaccination coverage exceeds 70%. All statistical analyses, including the creation of maps, were conducted using R programming language version 4.3.1^33^. The code that support the findings of this study are openly available and can be obtained from the following link: https://github.com/olatunjijohnson/RabiesPaper

### Ethics statement

The study protocol was approved by the Medical Research Coordinating Committee of the National Institute for Medical Research of Tanzania (NIMR/HQ/R.8a/Vol.IX/2019), the Institutional Review Board of the Ifakara Health Institute and the Tanzania Commission for Science and Technology (COSTECH) and was carried out in accordance with relevant guidelines and regulations. This research was done in compliance with the ARRIVE guidelines and regulations (https://arriveguidelines.org). All national and institutional guidelines for animal care have been followed throughout the study procedures. Dog vaccinations against rabies is the legal requirement in Tanzania.

## Results

During the fifth round of dog vaccinations held between October 2016 and January 2017, about 81, 000 dogs were vaccinated against rabies. The campaigns were conducted in 1,379 (68%) villages within the 25 districts of southeast Tanzania. Transect surveys were carried out in 1,287 of the 1,379 villages where dog vaccinations were implemented. During these surveys, approximately 10,474 dogs were counted, of which 7,053 were marked with collars, indicating they had been vaccinated.

### Campaign completeness

In the multivariable regression analysis, we found that geographical factors associated with campaign completeness are land cover, residence, poverty and elevation (Table 3). The Table shows the result for the odds calculated as exp($\beta$). For land cover, the result suggests that the odds of achieving vaccination completeness in areas classified as croplands were 1.75 times higher compared to forest, with a 95% CI ranging from 1.02 to 2.92. For residence, urban areas had significantly higher odds (8.82, 95% CI: 5.10—15.87) of achieving vaccination completeness compared to rural areas. Higher poverty levels were associated with increased odds of achieving vaccination completeness (3.11, 95% CI: 2.53—3.83). Higher elevations were associated with lower odds of achieving vaccination completeness (0.85, 95% CI: 0.75—0.95), indicating a negative relationship between elevation and vaccination completeness. We also found a spatial effect ($\lambda =0.61$).

**Table 3:** Results of the final multivariable logistic regression model predicting campaign completeness and vaccination coverage. HQ = District headquarters, VU = Vaccination units.

Intercept	Completeness estimate (95 CI)		Coverage estimate (95 CI)	
	Univariable	Multivariable	Univariable	Multivariable
		2.12 (1.85—2.43)		2.04 (1.95 – 2.15)
Land cover				
Forest	REF	REF	REF	REF
Shrubland & savannas			0.73 (0.28- 1.67)	
Croplands	1.56 (0.15—61.5)	1.75 (1.02 – 2.92)	0.69 (0.26—1.64)	
Wetlands			0.37 (0.03—5.05)	
Urban and build-up lands			0.53 (0.19—1.41)	
Residence (Urban)	1.64 (0.18—35.3)	8.82 (5.10 – 15.87)	0.68 (0.46—1.01)	0.73 (0.65 – 0.82)
Poverty index	1.05 (0.31—2.88)	3.11 (2.53 – 3.83)	1.14 (1.02 -1.28)	1.10 (1.06 – 1.14)
Protected (Yes)	1.41 (0.15—33.5)	–	1.55 (0.74 -3.55)	
Elevation	0.96 (0.33—3.04)	0.85 (0.75 – 0.95)	0.96 (0.86—1.08)	0.94 (0.91 – 0.97)
Area (in km.sq)	1.00 (0.40—5.31)	–	1.03 (0.93—1.21)	0.97 (0.94 – 0.99)
Population density	0.89 (0.36—3.45)	–	0.92 (0.83—1.01)	
Distance: HQ to VUs (in km)		–	1.16 (1.02—1.32)	–

### Vaccination coverage

Geographical factors found to be associated with vaccination coverage are: residence, area, poverty index and elevation (Table 3). Contrary to vaccination completeness, urban areas had lower odds of achieving vaccination coverage compared to rural areas (0.73, 95% CI: 0.65—0.82). Similar to vaccination completeness, higher poverty levels were associated with increased odds of achieving vaccination coverage (1.10, 95% CI: 1.06—1.14); and higher elevations were associated with lower odds of achieving vaccination coverage (0.94, 95% CI: 0.91—0.97). We further found that larger geographical areas had lower odds of achieving vaccination coverage (0.97, 95% CI: 0.94—0.99). We also found a strong spatial pattern ($\lambda =0.73$).

### Map of vaccination completeness and vaccination coverage

**Fig. 2:**
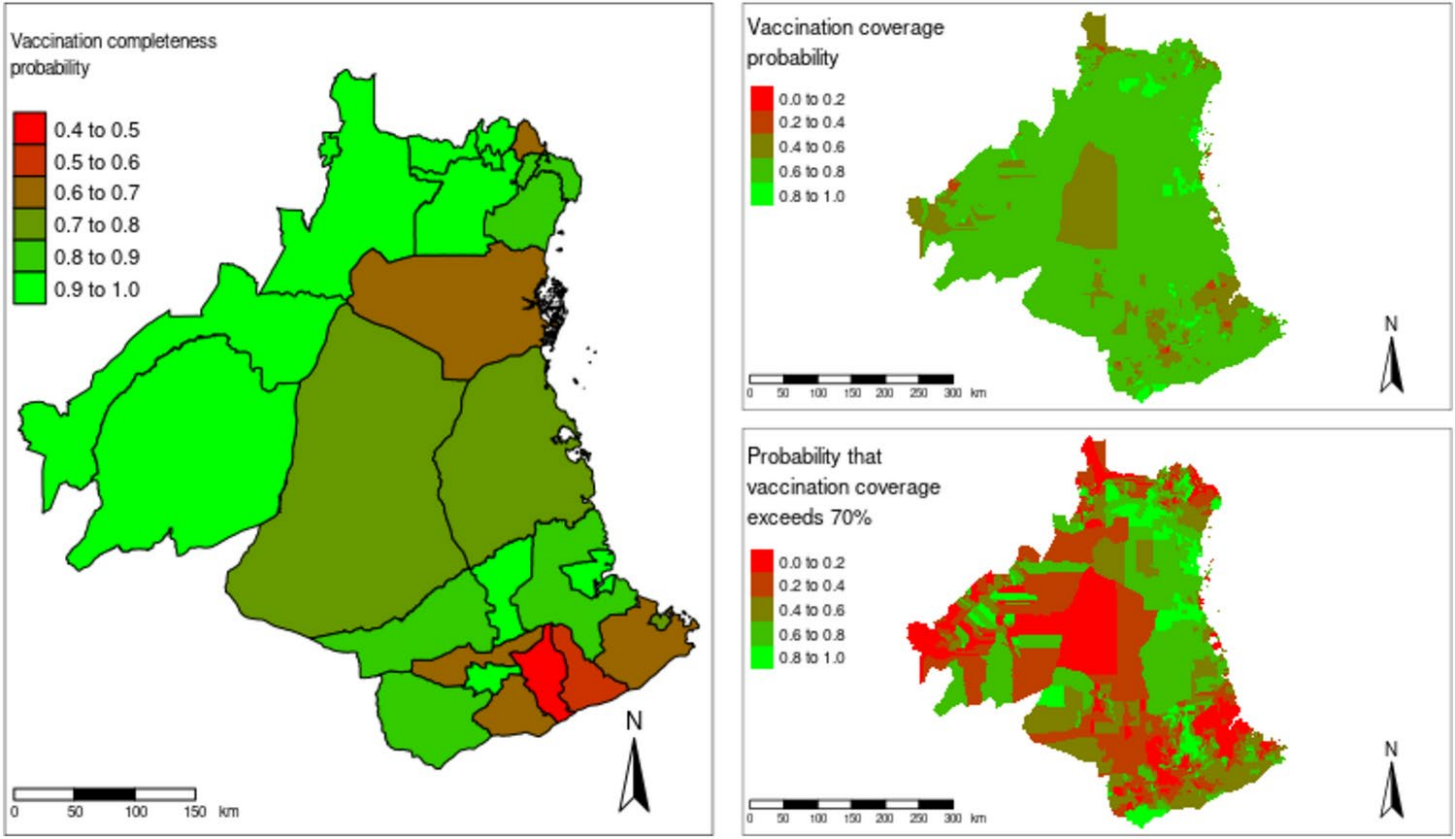
The maps show the probability of completeness modeled at district level (left panel), the coverage probability (topright panel) and the probability that village-level vaccination coverage exceeds 70% (bottomright panel) in south-eastern Tanzania. The shapefiles used in these figures were downloaded from the Diva GIS data portal (https://www.diva-gis.org/).

The resulting map from the prediction model showed higher campaign completeness (close to 100%) in districts with more experience delivering dogs vaccinations, such as Ulanga and Kilombero districts (Fig. 2). We also found that the campaign completeness is lower (< 50%) in districts with larger number villages particularly in rural districts. On the other hand, vaccination coverage is highly variable across all 25 districts (Fig. 2, topright). There is a high degree of heterogeneity in vaccination coverage within the districts, with some vaccination units having over 80% coverage and others having less than 20% coverage. To convey the uncertainty and also identify areas where the coverage could be improved, we map the exceedance probability, that is, the probability that the vaccination coverage exceeds 70% (Fig. 2, bottomright). We found that these probabilities was lower on the islands (located in the Indians oceans), as well as in highlands (mountainous areas), urban areas, and areas bordered to the protected areas.

## Discussion

This study attempts to characterize the influence of geographical factors on campaign completeness and vaccination coverage in Tanzania. While it is impossible to modify geographical factors, the study underscores the importance of learning logistical challenges caused by these geographical factors to improve the delivery of not only dog vaccinations but other drugs or vaccines to be delivered at scale.

### Campaign completeness

Our multivariable analysis shown that urban areas were 8 times more likely to achieve completeness than rural areas. We hypothesize that it was simpler to achieve campaign completion in urban settings compared to rural districts because vaccination unit was at ward level (district with most vaccination unit was Kibaha urban (n = 53) which is relative rural district) therefore urban district had fewer vaccination units compared to rural districts. The allocated budget was enough for urban districts to reach all vaccination units (all wards) as shown in Table 1. In addition, urban areas are smaller (and more densely populated) making it easier (transport vaccinators) to cover all vaccination points than in rural areas as well as being easier for dog owners to bring their dogs to vaccination points.

Our analysis also showed that probability of campaign completeness is higher in croplands areas compared to forested areas. The cropland areas have good access and transport networks to transfer people and crops compared to forest areas that are less populated and have poor transport networks.

### Vaccination coverage

Our final model indicated that poverty index had positive odds of achieving higher coverage. This is an interesting result as rural areas were dominated by poor people compared to urban areas. Rural areas that are historically known to own larger number of dogs were prioritized by livestock officers for vaccinations. We also found that large geographical areas had lower likelihood of achieving higher coverage. The protocol of delivery of dog vaccinations was one central point per village whereby dog owners voluntarily bring their dogs for parental vaccination. Locating one vaccination point per village regardless of the geographical size of the village made it hard for villagers located far away from vaccination points (normally located at the centres of the villages) to vaccinate their dogs. Previous studies observed that when vaccination points were located far away from their homes, many dog owners lacked interest in bringing their dogs for vaccinations^34–36^. This was confirmed in our study districts (Ulanga and Kilombero) through qualitative study which revealed that the owners did not take their dogs to long-distance vaccination sites^37^. Turn-out at vaccination points would be too low to vaccinate sufficient dogs to control rabies in large villages. Therefore, it recommended to increase vaccination points in these areas^37,38^.

Our findings showed that an increase in elevation reduced the odds of achieving adequate coverage. This could be associated with the topography whereby steep terrain made it difficult for both dog vaccinators to reach vaccination points (out of way) but also for dog owners to bring their dogs. Our previous work in two study districts (Ulanga and Kilombero) showed that campaign announcements were often focused solely around accessible routes, shops and the village offices making difficult for announcement to reach ‘*hard to reach*’ areas^37^. Our finding is in agreement with a previous study in Peru showed that steep slopes was identifies as a barrier to dog vaccinations^39^. Due to the challenging terrain in mountainous regions, reaching all dog-owning households requires a considerable vaccination effort, resulting in longer timeframes. Achieving higher evenness coverage in these areas necessitates a carefully coordinated vaccination strategy, potentially involving the deployment of additional vaccinators and additional vaccination points.

Our result found that urban areas had lower vaccination coverage compared to rural areas. This finding is inconsistent with study in Malawi that found a small difference in coverage between rural and urban areas, for example Blantyre urban achieved 85.5% coverage versus 82.8% achieved in Blantyre rural^36^. Our previous work showed that urban areas were not prioritized compared to rural areas^15^. Our findings mark an important addition that although it is easier to achieve campaign completeness in urban areas but it is not necessarily results in achieving higher coverage. An in-depth evaluation should be conducted focusing urban areas to investigate the barriers to dog vaccinations i.e., we speculate that probably urban areas were too busy to bring dogs for vaccinations. It is therefore important to prioritize urban areas for the effective vaccination campaigns through better advertising, better timing to accommodate urban residents’ schedules such as expand vaccination services beyond traditional vaccinations hours and conducting MDVs during weekends.

In this study, we found a lower probability of achieving campaign completeness in districts with more villages, particularly in rural districts. Campaign completeness was calculated as the percentage of villages where vaccination campaigns were conducted, i.e., the number of villages reached divided by the total number of villages in the district, multiplied by 100. The lower probability of achieving vaccination completeness in districts with more villages was likely due to logistical challenges. Districts with more villages required additional financial resources to cover all areas effectively. However, budget allocations did not account for the greater number of villages, leading to insufficient funds to reach every village. Our result showed that the budget allocated for dog vaccination did not reflect the reality. For example, Rufiji, Newala, Tandahimba and Lindi rural and Mtwara rural districts had lower campaign completeness (Fig. 2) and received less budget per village (Table 1). Rufiji district has multiple island villages that requires motorised boats to reach, with transport between the islands costly and not reliable. The allocated amount (10.5 million Tanzanian shillings). Consequently, logistical constraints, such as limited budget and resources, resulted in some villages being missed during the vaccination campaigns, thereby reducing the overall probability of achieving campaign completeness in these districts. Future budgeting should incorporate the size of geographical area to improve campaign completeness.

We used exceedance probability to identify areas where vaccination coverage could be improved, our results showed that urban areas, small islands (in the Indian ocean), highlands (mountainous areas), and areas bordering protected should be target as priority areas. Specialized approaches tailored to specific needs is required to improve coverage in these areas. For example, allocating additional budget or additional vaccinators in the hard to reach areas whereas social mobilization and community engagement about the importance of dog vaccinations is required in urban areas.

Our previous work showed difficulties in achieving higher coverage in Mahenge plateau and Udzungwa mountains in the northern parts of Ulanga and Kilombero districts, where the terrain makes these areas difficult to reach to vaccination point^37^. The lack of road infrastructure and the mountainous terrain are challenges for central point vaccinations. The combination of distance, and lack of transport made it difficult to achieve 100% campaign completeness in mountainous areas. We recommend an extra budget and an extra vaccination points in these areas. Lesson learnt that higher coverage was achieved when an extra vaccination point were added in Machipi village in Kilombero district (bordering the Udzungwa mountains). We also recommend house-to-house dog vaccination approach in these mountainous areas and other hard to reach areas. We recommend supporting local volunteers from these communities to vaccinate dogs. For example, a community-based continuous mass dog vaccination (CBC-MDV) approach using Rabies One Health Champions in Mara region in Tanzania has been shown to be effective^40^. Previously the use of Community Animal Health Workers (paravets) were successful in delivering dog vaccinations using house-to-house approach in Masaai areas in Tanzania^9^. The Community-directed vector control to supplement mass drug distribution for neglected tropical diseases were documented to be successful and the cornerstone strategy for the African Programme for Onchocerciasis Control^41^. It is important to maintain a strong focus on reaching people most in need and delivering MDVs regardless of access constraints, rather than on delivering MDVs in the easiest-to-reach areas.

We propose implementing dog vaccination campaigns in villages situated adjacent to protected areas, game reserves, forests, and national parks to safeguard wildlife from rabies. Dog vaccinations in these areas will act as a buffer zone to protect wildlife, domestic dogs serve as reservoirs for rabies, posing a risk of spillover to other species^42^. On other hand, our results showed that large geographical areas exhibit a reduced likelihood of achieving vaccination coverage exceeding 70%. However, most of the large districts (such as Newala, Ulanga, Kilwa etc.) encompass protected areas or forest reserves, for example, 75% of the landmass of Ulanga district is protected areas (no human settlement of human activities) which facilitates the logistical implementation of MDV campaigns in only 25% of the district landmass.

### Study limitation

Other contextual factors could have impacted vaccination campaign completeness and coverage that were not investigated in this study. Factors such as lack of awareness that the campaign was taking place, not being able to find or handle their dog, the degree of community trust or acceptability towards the dog vaccinations, as well as conflicting schedules during vaccinations that have been reported to the barriers to dog vaccinations were not investigated^37^. We used INLA as our chosen analysis method. While INLA is a powerful tool for Bayesian inference, alternative methods such as Markov Chain Monte Carlo (MCMC) could potentially offer advantages due to their ability to provide a more thorough exploration of the parameter space. Dog vaccinations were conducted over five rounds. In this study, we focused solely on the fifth round of vaccination, as it had the most complete data, which may have influenced the results observed. We acknowledge this as a limitation of the study.

### Future work

A potential area of future work research could incorporate demographic and geographical factors together with bite incidence data to determine the width and location of vaccination zones that could most effectively interrupt transmission and maintain rabies-free areas. In future we hope to use data on rabies transmission for disease transmission models, to explore the effectiveness of dog vaccination interventions. Initially, our transect data were paper-based, which limited our ability to perform real-time data analysis and respond rapidly with remedial (mop-up) campaigns in areas with low vaccination coverage (poor-performing villages). This approach hindered our ability to correlate the modeling results with the actual situation effectively. Conducting transect surveys using mobile phone applications could help to assess completeness and coverage rapidly and inform poor-performing areas where mop up (remedial) campaigns could be implemented^43,44^.

## Conclusions

Our study has identified geographical barriers to campaign completeness and vaccination coverage. Landscapes are not homogeneous, therefore it is important to design and implement targeted vaccination campaigns to achieve completeness and higher vaccination coverage. Multisectoral collaboration is essential to overcome identified barriers. For example, the involvement of the wildlife sector in controlling rabies in neighboring areas (buffer zones) plays a crucial role in mitigating the spread of the rabies. Additionally, involvement of transport authorities to improve road networks in hard-to-reach areas facilitates the efficient distribution of vaccines and ensuring that remote communities have access to dog vaccinations. Furthermore, leveraging human health cold chain infrastructure for storing dog vaccines in remote, hard-to-reach locations is of paramount importance. Overall, this study underscores the significance of addressing geographic disparities in delivering dog vaccinations to achieve the 2030 national target for eliminating dog-mediated rabies in Tanzania.

## Data Availability

The code that support the findings of this study are openly available and can be obtained from the following link: https://github.com/olatunjijohnson/RabiesPaper.
